# Lignin and Cellulose Blends as Pharmaceutical Excipient for Tablet Manufacturing via Direct Compression

**DOI:** 10.3390/biom9090423

**Published:** 2019-08-28

**Authors:** Juan Domínguez-Robles, Sarah A. Stewart, Andreas Rendl, Zoilo González, Ryan F. Donnelly, Eneko Larrañeta

**Affiliations:** 1 School of Pharmacy, Queen’s University Belfast, 97 Lisburn Road, Belfast BT9 7BL, UK; 2 Instituto de Cerámica y Vidrio, CSIC, Calle Kelsen, 5, 28049 Madrid, Spain

**Keywords:** lignin, microcrystalline cellulose, pharmaceutical excipients, direct compression, tablets, tetracycline

## Abstract

Extensive efforts are being made to find alternative uses for lignin (LIG). In the present work the use of this biopolymer as excipient to prepare tablets was studied. For this purpose, LIG was combined with microcrystalline cellulose (MCC) and used as excipients to prepare directly compressed tablets containing a model drug, tetracycline (TC). The excipients contained different concentrations of LIG: 100%, 75%, 50%, 25% and 0% (*w*/*w*). Two different compression forces were used (two and five tonnes). When formulations were prepared using LIG as the only excipient, tablets were formed, but they showed lower densities and crushing strength than the ones obtained with only MCC or LIG/MCC blends. Moreover, tablets prepared using five tonnes of compression force showed TC releases ranging from 40% to 70% of the drug loading. On the other hand, the tablets prepared using two tonnes of compression force showed a faster and more efficient TC release, between 60% and 90%. The presence of LIG in the tablets modified significantly the release profile and the maximum amount of TC released. Finally, a DPPH (2,2-diphenyl-1-picrylhydrozyl) assay was performed to confirm that the presence of LIG provided antioxidant properties to the formulations. Accordingly, LIG has potential as a pharmaceutical excipient.

## 1. Introduction

Tablets are the most commonly used pharmaceutical dosage form [1]. They are relatively simple to manufacture, show good physical stability and they are extensively accepted by patients [1,2]. Direct compression is the preferred method of tablet preparation. This method involves the tableting of a mixture of ingredients without preliminary agglomeration or granulation processes [3]. This method presents advantages over other tableting methods such as wet granulation as it requires shorter processing times, fewer excipients and reduced stability risk during processing [4]. Different pharmaceutical excipients can be used for direct compression, including a wide range of polymers [5,6,7]. These polymers include synthetic macromolecules, such as poly (vinyl pyrrolidone) or poly (acrylic acid), and natural polymers, such as cellulose [5].

Cellulose is one of the most important excipients used in tableting due to its excellent binding properties in the dry state [3]. Moreover, cellulose is the most abundant natural polymer on Earth [3,8,9]. This biopolymer is present in plant cell walls and, accordingly, is a renewable raw material [3,5,8]. However, in addition to cellulose and its derivatives, the majority of the excipients used in solid oral dosage forms are synthetic polymers [10]. The development of green and renewable biopolymers that replace synthetic polymers for material production has attracted significant attention [11,12,13,14,15]. Accordingly, there is a need to find new renewable polymers that can be used for pharmaceutical applications [14,15,16,17]. Considering that the pharmaceutical excipient market is expected to be worth 8.53 USD billion by 2023 [18], extensive efforts have been made to develop new excipients for tablet preparation [19,20]. A good renewable and cost-effective candidate for this purpose is lignin (LIG).

LIG is a biopolymer present in the cell walls of vascular plants formed by randomly crosslinked networks of methoxylated and hydroxylated phenylpropane [11,21,22,23]. This compound provides mechanical protection to the plant. Moreover, LIG protects the plants from external biological and chemical stresses as it possesses antioxidant and antimicrobial properties [24,25,26,27,28]. LIG is one of the most abundant polymers on Earth, second after cellulose [11,22,29,30]. The main difference between cellulose and LIG is that the latter remains relatively unexploited [11,31]. The majority of the close to 70 million tonnes of LIG produced during cellulose extraction by the paper industry is burnt as low grade fuel or just discarded as a waste [11,32]. Less than 2% of the total amount of LIG produced is reused to manufacture specialty products [11]. Due to its abundance and added value properties (antioxidant and antimicrobial activities), LIG has considerable potential to be used in new functional and green materials.

During the last decade, researchers have made extensive efforts to develop new LIG-based materials [11,33]. This biopolymer has been used in a wide variety of applications such as antimicrobial agent, antioxidant additive, UV protective agent, hydrogel forming molecule, nanoparticles component or binder in lithium batteries among others [34,35,36,37,38,39,40,41]. However, the use of LIG as excipient for pharmaceutical formulations is scarce, and only a few studies describe its use [19,42,43]. Accordingly, more work is necessary to complement the findings described in these papers to fully understand the potential of LIG as pharmaceutical excipient.

In the present work, we propose the use of LIG as an excipient for direct compression in the preparation of drug containing tablets. For this purpose, a model drug was selected, tetracycline (TC), and was combined with LIG to prepare tablets. Additionally, LIG was combined with microcrystalline cellulose (MCC) to prepare different types of tablets. The tablets were characterised by evaluating their crushing strength, homogeneity of content, morphology, wettability, antioxidant properties and drug release.

## 2. Materials and Methods 

### 2.1. Materials

The LIG used was BioPiva 100, a softwood Kraft LIG purchased from UPM (Helsinki, Finland). Prior to its use, LIG was milled using a mortar and pestle in order to remove the existing lumps and subsequently placed into the oven at 60 °C for 24 h to remove excess moisture. Chemical characterisation and molecular mass of the used LIG sample was kindly provided by the supplier. Klason LIG content (TAPPI T 222 om-02) is around 92% of the dry matter and acid-soluble LIG (TAPPI UM 250) is around 4% of the dry matter. The sum of Klason LIG and acid-soluble LIG (96%) is commonly considered the value of total LIG content. On the other hand, the total amount of carbohydrates (SCAN-CM 71:09) accounts for around 2% of the dry matter and the content of inorganic particles (internal method, 700 °C) accounts for around 1% of the dry matter. Finally, the molar mass of this LIG sample is between 5000–6000 Da. These values are according to those found in other softwood Kraft LIG samples [38,44]. MCC, Avicel PH 102, was acquired by IMCD UK Limited (Sutton, UK). Finally, the model drug employed in this study, TC, was purchased from Honeywell Fluka™ (Leicestershire, UK).

### 2.2. Powder Characterisation

The morphology of MCC and LIG powder was evaluated by using scanning electronic microscopy (SEM). Pictures were taken under vacuum using a Hitachi TM3030 environmental SEM (Tokyo, Japan). 

Malvern Mastersizer 3000 instrument (Malvern, UK) fitted with an Aero S dry dispersion unit was used to determine the particle size distributions of LIG and MCC. Approximately 1 g of each excipient was weighed and added to the general tray. Using an air pressure of 1 and 1.5 bar for MCC and LIG, respectively, and a feed rate of 40% to provide a reasonable flow of powder into the instrument. Three measurements were performed for each sample to give an estimate of the variability about the measurement.

To determine bulk and tapped densities, approximately 50 g of each granule sample was poured into a 100 cm^3^ cylinder, and the volume was measured. Immediately after, the powder mass was tapped 50 times, and the volume was measured again [45]. Bulk and tapped densities were calculated using Equations (1) and (2) respectively and used to determine the Hausner ratio and the Carr’s compressibility index using Equations (3) and (4), respectively. The experiments were repeated three times.

(1) $$Bulk\;Density=\;\frac{Sample\;Mass}{Volume}$$

(2) $$Tapped\;Density=\;\frac{Sample\;Mass}{Tapped\;Volume}$$

(3) $$Hausner\;Ratio=\;\frac{Tapped\;Density}{Bulk\;Density}$$

(4) $$Carr\;Index=\;\frac{Tapped\;Density-Bulk\;Density}{Tapped\;Density}$$

The Brunauer–Emmet–Teller (BET) method was applied to calculate the specific surface area (S_BET_) and pore size of the MCC and LIG from the desorption curve. The samples were degassed under nitrogen flow, heated to 90 °C for 3 h and then to 150 °C for 24 h until they reached constant weight. These powder properties were analysed by means the nitrogen adsorption isotherm, using a Micrometrics TriStar II porosimeter.

In order to evaluate the compactability of MCC and LIG powders, the Kawakita model was used (Equation (5)) [46].

(5) $$\frac{P}{C}=\;\frac{P}{a}+\frac{1}{b}$$

The porosity of the samples was calculated using the Equation (6) [46].

(6) $$Porosity=\;1-\frac{Aparent\;Density}{True\;Density}$$

where apparent density is the density of the powder out-of-die after applying a determined pressure (*P*). The theoretical densities of MCC and LIG were measured by using pycnometric density method (AccuPyc 1330, Micromeritics Instrument, Norcross, GA, USA).

This equation correlates the degree of volume reduction (*C*) with the applied pressure (*P*). The Kawakita parameters (*a* and *1*/*b*) are calculated after plotting the values of *P*/*C* vs. *P.* The values of *C* can be calculated using Equation (7) [47].

(7) $$C=\;1-\frac{Bulk\;Density}{Apparent\;Density}$$

Apparent density is the density of the powder out-of-die after applying a determined pressure (*P*). Accordingly, in order to calculate *C*, specific amounts of powder were compressed at different pressures, and the density of the resulting tablets was calculated (Apparent density).

Finally, Heckel equation (Equation (8)) was used to evaluate the densification of LIG and MCC powders under pressure [46].

(8) $$Ln\;\left(\frac{1}{1-D}\right)=KP+A$$

where *D* is the relative density of the tablet at applied pressure *P*, and *K* is the slope of the straight-line portion of the Heckel plot. Moreover, 1/*K* gives mean yield pressure, *P*_γ_, that is related to the yield strength of the material. The parameter A gives densification of the powder due to initial particle rearrangement (Da). Da is calculated from Equation (9).

(9) $$D_{a}=1-e^{-A}$$

### 2.3. Tablet Manufacture

Cylindrical tablets of LIG and MCC with a diameter of 13 mm, a mass of approximately 500 mg and thicknesses of 2.56–2.85 mm were produced by using a manual hydraulic press (Specac Limited, England, UK) and by applying two different compression forces (2 or 5 tonnes) for 1 min, for each tablet formulation. Prior to compression the different excipients and the tetracycline were properly mixed in a 50 mL Falcon tube using a vortex mixer. For each tablet, tetracycline represented 50% of the total mass (250 mg), while the remaining 50% consisted of different proportions of the other constituents. Subsequently, the die was filled manually.

### 2.4. Tablet Characterisation

The properties of the produced tablets were assessed by applying test contained in the European Pharmacopeia 8.0. Tablet crushing strength (hardness) measurements were performed on at least three tablets for each different composition and applied compression force using a Tablet tester 5Y (Copley Scientific Limited, Nottingham, UK). In order to obtain the tensile strength equation 10 was used [48].

(10) $$Tensile\;Strenght=\;\frac{2P}{\pi Dt}$$

where *P* is crushing strength, *D* is the tablet diameter and *t* is the tablet thickness.

For the mass uniformity, a minimum of 6 tablets were randomly selected and weighed. The average mass was then calculated, and the percentage deviation of each individual mass was determined.

The morphology of the produced tablets was evaluated by using scanning electronic microscopy (SEM). Pictures were taken under vacuum using a Hitachi TM3030 environmental SEM (Tokyo, Japan). A Leica EZ4 D digital microscope (Leica, Wetzlar, Germany) equipped with florescence filters (excitation 440–460 nm and emission 500 nm) (Nightsea, Lexington, MA, USA) was used to evaluate the distribution of TC within the tablets. Additionally, in an attempt to better understand the behaviour of the used excipients (LIG and MCC) in an aqueous medium, these materials were soaked in deionised water for 30 min and observed in a Leica EZ4 D digital microscope (Leica, Wetzlar, Germany).

The influence of the different excipient proportions employed on the contact angle of deionised water with the surface of the dry tablets was also evaluated using an Attension Theta equipment (Attension Theta, Biolin Scientific, Gothenburg, Sweden). OneAttension software analysed results to give an indication of the wettability of the surface. For this purpose, unloaded tablets (without tetracycline) applying a compression force of 5 tonnes were manufactured. The experiment was performed in triplicate.

### 2.5. Dissolution Testing

Dissolution testing of tablets was conducted according to the test method 2.9.3. of the European Pharmacopeia 8.0 by using a CALEVA 8ST dissolution tester (Caleva Process Solutions Ltd. Dorset, UK). The test was performed by placing a tablet in each vessel which was filled with 900 mL of distilled water and kept at a temperature of 37 °C. The paddle speed was adjusted to 75 rpm. The percentage of the released tetracycline was evaluated at defined time points using a UV–vis plate reader (PowerWave XS Microplate Spectrophotometer, Bio-Tek, Winooski, VT, USA) at a wavelength of 363 nm and after each measurement the medium was replaced with fresh media. Each manufactured batch was assayed in triplicate. The concentration of the dissolved tetracycline was calculated based on a previous calibration curve (from 0 to 0.03125 mg/mL) at a wavelength of 363 nm.

### 2.6. LIG-Microcrystalline Cellulose Antioxidant Activity

DPPH (2,2-diphenyl-1-picrylhydrozyl) (Sigma Aldrich; Dorset, UK) radical was employed to measure the antioxidant activity of the produced tablets based on the radical scavenging property of the LIG [26,49]. For this purpose, LIG, MCC, and their blends, according to the different tablet compositions, were dissolved in a dioxane (Sigma Aldrich; Dorset, UK)/water (90:10, vol/vol) solution with a concentration of 400 mg/L. Additionally, in order to assess the effect of the tetracycline on the antioxidant properties, a mixture containing LIG and MCC in a 1:1 ratio as well as tetracycline in the same proportions as the produced tablets was also dissolved in a dioxane/water (90:10, vol/vol) solution with a total concentration of 800 mg/L. On the other hand, a solution of 50 mg/L of DPPH in a dioxane/water (90:10, vol/vol) was made. Then, 70 µL of each mixture were mixed with 230 µL of the DPPH solution in a 96 well plate and the concentration of DPPH radicals were monitored at 517 nm in triplicate using a UV–vis plate reader (PowerWave XS Microplate Spectrophotometer, Bio-Tek, Winooski, VT, USA). A control sample of 50 mg/L of DPPH was also evaluated. The radical scavenging activity (DPPH inhibition) was calculated as Equation (10) [26].

(11) $$DPPH\;inhibition=\;\frac{A_{0}-A_{t}+A_{B}}{A_{0}}$$

where *A*_0_ = absorbance at 0 min; *A_t_* = absorbance at time *t*; and *A_B_* = absorbance of the blank solution (equivalent solution with no DPPH).

### 2.7. Statistical Analysis

All data presented in this paper were expressed as mean ± standard deviation. Results were compared using a paired, two-tailed Student’s *t*-test when comparing two means. When more than two means were compared, a One-Way Analysis of Variance (ANOVA) and Tukey’s HSD post-hoc test were used. In all cases, *p* < 0.05 was the minimum value considered acceptable for rejection of the null hypothesis.

## 3. Results

### 3.1. Powder Characterisation

**Figure 1:**
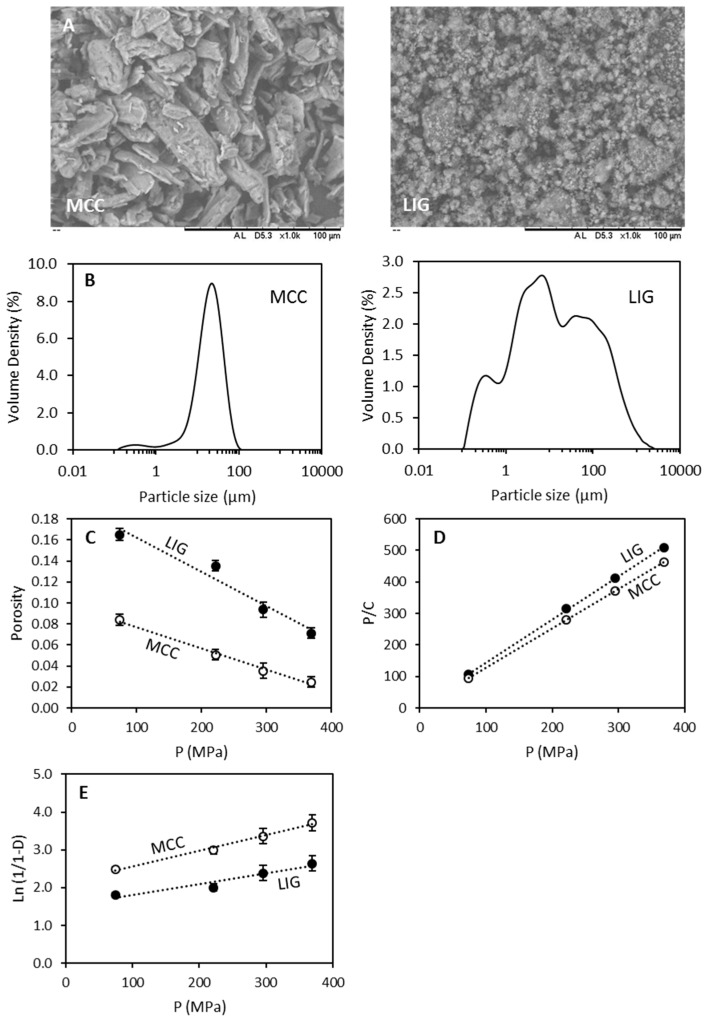
SEM images showing the morphology of microcrystalline cellulose (MCC) and lignin (LIG) powder (**A**). The scale bar in all cases represents 100 µm. Particle size distribution for MCC and LIG powders (**B**). Porosity of LIG and MCC as a function of the pressure (**C**) Kawakita plots for LIG and MCC powders (**D**). Heckel plots for LIG and MCC (**E**).

SEM microscopy was used to evaluate the morphology of the compounds used to prepare the tablets. The morphology MCC and LIG powders can be seen in Figure 1A. LIG powder presented a smaller particle size than MCC. This was confirmed by particle size measurements (Figure 1B). It is noticeable that MCC showed a monodisperse particle size distribution. This size distribution showed an average particle size of around 25 µm. Moreover, SEM images showed a relatively homogeneous particle size distribution for MCC with particles sizes ca. 25 μm (Figure 1A). This is consistent with the particle measured particle size distribution. On the other hand, LIG powder showed three main size populations. The majority of the particles presented a size around 6 µm. Moreover, some aggregates can be observed showing a size around 60 µm. Finally, the smaller population showed a size of around 0.3 µm. Again, these results were consistent with the SEM images that showed smaller particles for LIG and some aggregates (Figure 1A). The particle size displayed by LIG shows smaller granule size than the one reported by Penkina et al. for equivalent LIG (softwood Kraft LIG) that showed an average particle size of around 350 µm [19]. This factor is important as it has been reported that powders with smaller particle size distribution yield tablets with reduced crushing strength [50]. Moreover, the obtained particle sizes for LIG are smaller than the ones obtained by Pishnamazi et al. for Organosolv lignin ca. 100 µm [42,43].

The bulk density and tapped density of MCC and LIG powder were measured (Table 1). The obtained bulk and tapped densities of MCC are similar to the ones reported previously by other authors [51,52]. On the other hand, LIG showed higher bulk and tapped densities than MCC (*p* < 0.05). As the use of LIG as pharmaceutical excipient has been scarcely studied before, there are no reports of the bulk densities or tapped densities of LIG. 

In order to evaluate the flow properties of LIG, Hausner ratio and Carr index were calculated (Table 1). Interestingly, the values of these parameters showed no significant differences for MCC and LIG (*p* > 0.05). The obtained values for Hausner ratio were between 1.12 and 1.18, and the Carr index values were between 11 and 15. Powders that fall in this category are considered to have “good” flow properties [53]. Accordingly, LIG shows promising flow properties to be used as pharmaceutical excipient.

Table 1 shows the pore size distribution of LIG and MCC powders and its BET measured surface areas. The surface areas obtained for MCC are in line with the values previously reported [54,55]. The pore sizes obtained for both materials are similar, however the specific surface area of LIG is higher than the one obtained for MCC. This is consistent with the results obtained for the particle size. LIG powder has smaller particle size and accordingly it presents higher surface area. The porosity of LIG and MCC powders was calculated (Table 1). MCC showed a larger amount of void space between particles than LIG. However, when MCC powder was compressed the resulting MCC tablets showed lower porosity than LIG tablets. Accordingly, this suggest that LIG is not as compactable as MCC.

Kawakita and Heckel equations were used to study the compressibility and the densification of LIG and MCC powders. Figure 1D shows the Kawakita plots for both compounds. By applying Kawakita equation to this data the Kawakita parameters (*a* and *1*/*b*) for MCC and LIG were obtained (Table 2). The parameter *a* represents the value of initial porosity [46]. Moreover, this value mathematically represents the degree of compression or engineering strain at infinite pressure. It also represents to the total portion of volume that is reducible at maximum pressure. The obtained values of *a* for MCC and LIG suggest that both materials have similar porosity. This is consistent with the pore size values measured using BET (Table 1). Moreover, this suggest that both materials have similar degree of compressibility at infinite pressure. On the other hand, the *1*/*b* parameter represents the pressure required to compress the powder to half of the total volume reduction [46]. Additionally, *1*/*b* is proposed to be related to the yield strength and plasticity of the material [46]. MCC and LIG showed different values for the *1*/*b* parameter. These values suggested that MCC requires less pressure to be compacted to the same degree than LIG. Moreover, these values suggest that LIG has a higher yield strength and plasticity than MCC. On the other hand, Heckel parameters showed that MCC presented higher densification and hence lower Py values. Moreover, D_a_ values showed that the densification due to initial particle rearrangement is also higher for MCC than for LIG. Accordingly, MCC is easier to densify than LIG. This is consistent with the obtained values of porosity at different pressures (Figure 1C).

### 3.2. Tablet Morphology and Characterisation

**Figure 2:**
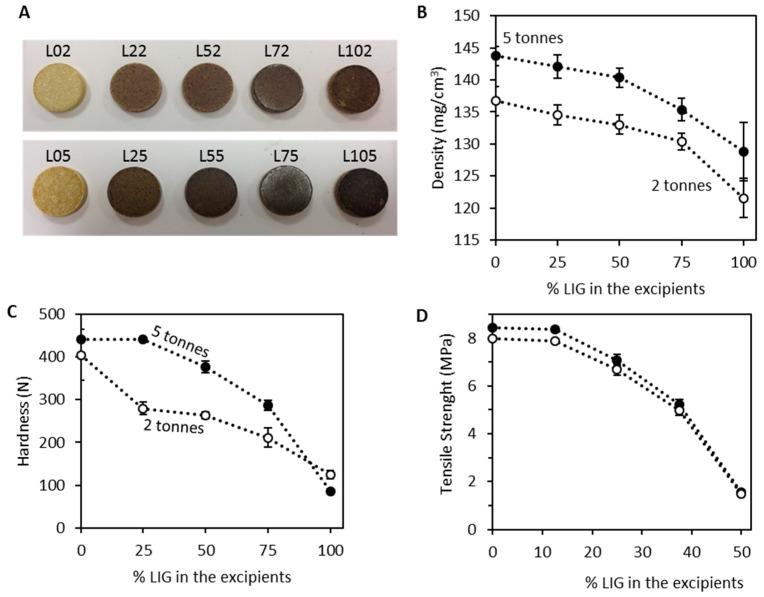
Images of all the tablets prepared (**A**). Density (**B**), hardness (**C**) and tensile strength (**D**) of the tablets as a function of the percentage of LIG used as excipient.

Figure 2A shows a picture of all the TC containing tablets prepared using LIG and MCC as excipients. All the blends were successfully compressed to form tablets using two different compression forces: 2 and 5 tonnes. It is noticeable that tablets with higher LIG content present a darker colour due to the colour of LIG. Moreover, Table 3 shows that as expected, tablets prepared using 5 tonnes of compression force showed a lower thickness (*p* < 0.05) and, accordingly, a higher density (*p* < 0.05). This can be seen in Figure 2B. Also, LIG content in the formulations has a strong influence in the density of the resulting formulation. Tablets prepared using higher amounts of LIG showed lower thickness and density. However, the differences in the densities of tablets made with less than 50% of LIG in the excipients did not show significant differences between them (5 tonnes: 0%/25% LIG, *p* = 0.783; 0%/50% LIG, *p* = 0.098; 25%/50% LIG, *p* = 0.674; 2 tonnes: 0%/25% LIG, *p* = 0.326; 25%/50% LIG, *p* = 0.609). These findings are in line with the results obtained from the Kawakita analysis of the powders. LIG showed lower compactability than MCC. Accordingly, LIG containing tablets will present lower densities than the ones containing higher amounts of MCC.

Tablets prepared using only LIG as excipient showed a more heterogeneous mass distribution (*p* < 0.05). On the other hand, there were no significant differences in the mass homogeneity for the other tablets (*p* > 0.05). They showed higher percentage of variability (see mass uniformity in Table 3) when compressed using 2 or 5 tonnes. When the only excipient used was LIG, the resulting tablets were more brittle and more prone to lose weight. This was consistent with the density values (Figure 2B) that drops when MCC is not used in the formulations. This is especially noticeable when using 2 tonnes as compression force. Additionally, tablet crushing strength followed a similar pattern (Figure 2C). Tablets displaying a value of 441 N (L05 and L25) of crushing strength were not fractured during the test as this is the maximum value that the testing equipment can measure. The force required to fracture the tablets is higher for the formulations containing lower amounts of LIG as excipient. When the tablets were compressed using 5 tonnes the resulting tablets are harder (*p* < 0.05). Interestingly, formulations prepared using 100% of LIG or MCC showed similar crushing strength independently of the compression forces. Moreover, the statistical analysis showed no significant differences in crushing strength for these two types of tablets (*p* > 0.05). On the other hand, when the tablets were prepared using blends of MCC and LIG as excipients the crushing strength of the tablets is reduced (*p* < 0.05). Tablets containing 25% and 0% (only MCC) of LIG in the excipients compressed using 5 tonnes showed equivalent crushing strength (*p* > 0.05). Similarly, tablets containing MCC/LIG blends prepared using 2 tonnes of compression force displayed lower crushing strengths. Finally, tablets containing 25%, 50% and 75% of LIG in the excipients compressed using 2 tonnes showed no significant crushing strength differences (*p* > 0.05). The crushing strength of the tablets is influenced by the geometry, as tablets showed different thicknesses depending on the compositions. Tensile strength (Figure 2D) can be used to compare different geometries. The results suggested that as expected when LIG content increased the tensile strength of the tablets is reduced significantly (*p* < 0.05).

As described before, the addition of LIG to the tablets reduced tablet density. Accordingly, the structure presented a less compact structure. This explains why LIG containing tablets displayed lower crushing strengths than tablets prepared using only MCC as excipient.

As described previously, Penkina et al. used LIG to prepare tablets before. These tablets presented slightly higher resistance to fracture [19]. However, in this work the tablets were prepared using pure LIG, and they did not contain any drug like in the present work. On the other hand, Pishnamazi et al. described the use of lignin as excipient to prepare tablets containing acetylsalicylic acid [43]. Interestingly, these tablets displayed crushing strength close to 10 times lower (ca. 40 N) than the ones described in the present manuscript. However, these tablets contained lubricant and disintegrant (magnesium stearate and croscarmellose sodium).

**Figure 3:**
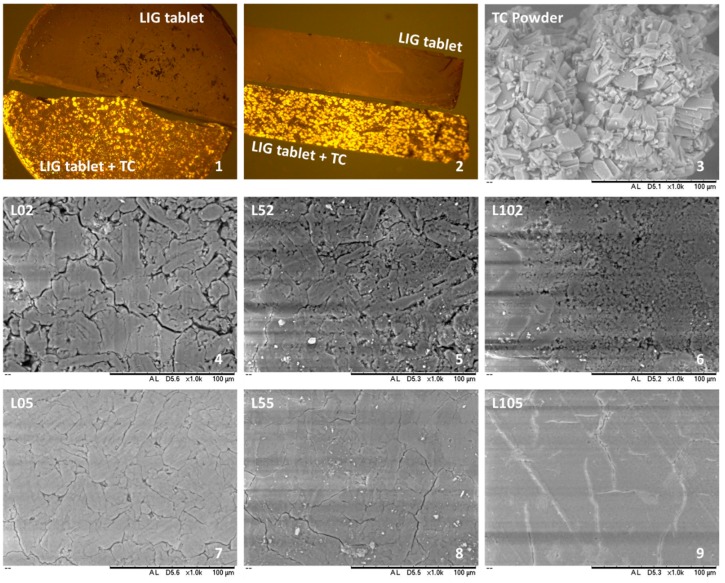
Fluorescence microscope image of tablets made of LIG with and without TC (**first two panels**). SEM images showing the morphology of TC powder and the surface of the tablets prepared (**panel 3 to panel 9**). The scale bar in all cases represents 100 µm.

Microscopy was used to evaluate the morphology of the tablets (Figure 3). First of all, fluorescence microscopy was used to evaluate the distribution of TC within the tablets. Figure 3 shows TC emitting fluorescence within a tablet this indicates that TC is uniformly distributed within the matrix. Moreover, the morphology of the tablets was observed using SEM (Figure 3); it is noticeable that tablets prepared using 5 tonnes as the compression force showed a more compact structure than when compressed using 2 tonnes. The latter showed a more noticeable interface between particles. Moreover, tablets prepared using LIG as the only excipient (L102 and L105) showed an apparently a more compact structure. However, by having a look at the previous results, these tablets presented a higher thickness and a lower density. Accordingly, this more compact appearance can be attributed to the smaller particle size displayed by LIG powder (Figure 1). The same effect can be observed for L52 and L55 shows an apparent more compact structure than L02 and L05 while showing lower densities.

Penkina et al. studied the mechanical properties of LIG powder when compressed, reporting that MCC showed better consolidation and deformation under compression values than LIG from different sources [19]. These results are consistent with the results presented here, showing that the inclusion of LIG in the formulations reduced tablet crushing strength and density. When pure LIG was used, there was a considerable decrease in tablet crushing strength and densities. However, when LIG/MCC blends were used as excipients they showed crushing strength (resistance to crushing) values higher than 200 N. These values were lower than the ones obtained for tablets formulated using MCC as binder. Nevertheless, LIG present other properties that can provide added value to the final product, such as antimicrobial and antioxidant properties [24,25,26,27,28]. 

### 3.3. Tetracycline Release from LIG/MCC Tablets

**Figure 4:**
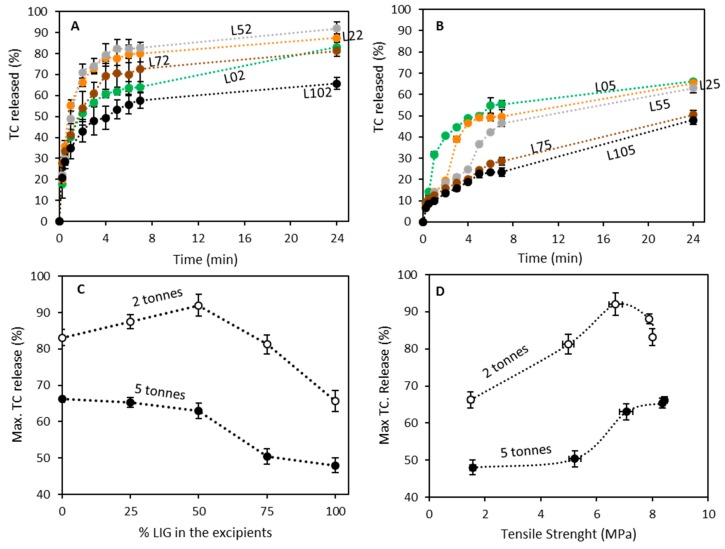
Tetracycline release profiles for LIG/MCC tablets prepared using 2 (**A**) and 5 (**B**) tonnes of compression force. Maximum TC release as a function of: the percentage of LIG used as excipient (**C**) and the tensile strength of the tablets (**D**).

The influence of LIG in the release profile of the formulation was studied. Figure 4A,B shows the drug release profile of the LIG/MCC tablets. Tablets prepared using 2 tonnes of compression force (Figure 4A,C) showed TC releases ranging from 66% to 91% of the drug loading. The formulations prepared using LIG as the only excipient (L102) showed the slower release profile. When MCC is added to the formulations a higher TC release was observed. The maximum amount of TC released was obtained for L52 (Figure 4C). However, the difference maximum TC release obtained for L52 and L22 is not statistically significant (*p* = 0.329). When the percentage of LIG starts to be higher than the MCC, one TC release start to decrease (*p* < 0.05).

When the compression force was increased, the resulting tablets displayed a lower amount of TC release (Figure 4B,C) (*p* < 0.05). This can be easily explained by having a look at the previous results. Tablets prepared using 5 tonnes of force showed higher density and crushing strength (Figure 2B,C). Accordingly, tablets cannot be completely disintegrated during the release. These tablets showed a swollen structure with an evident mass loss. In this case a maximum in the release profiles cannot be observed when LIG and MCC are used as excipients at a 1/1 ratio. Tablets containing 0%, 25%, or 50% of LIG did not show any significant difference in the maximum TC release (*p* > 0.05). However, when the content of LIG in the excipients is higher than 50% TC release is affected and reduces (*p* < 0.05). The max TC released by tablets prepared containing a 75% or 100% of LIG in the excipients can be considered equivalent (*p* = 0.937).

Pressure totally modifies the release profile. The percentage of release is not the only factor that needs to be considered. Figure 4A,B show that the release profiles obtained when the tablets were compressed using a compression force of 2 tonnes are all similar. However, tablets prepared using a higher compression force showed a different release profile. The tablets prepared using MCC as excipient (L02 and L05) showed similar release profile. The only difference is that L05 displayed a lower TC release due to the higher compression. L05 showed higher density and crushing strength (Figure 1B,C), and this explains why the tablets did not disintegrate as easily as L02.

When LIG is added to the tablets, the release profile shows a “jump” in the release profile at certain time points that was not observed for MCC only containing tablets. This “jump” can be observed at later times for formulations containing higher amounts of LIG. Finally, L105 did not show any “jump” in the release profile. This formulation showed a sustained but incomplete release curve. During the release, some formulations displayed tablet fracture (the tablet was separated into two or more pieces). This fracture was mainly observed only in formulations that contains MCC as excipient. Accordingly, this jump can be associated with this fracture.

The long release profiles obtained suggest that this type of tablets will be suitable for sustained release. Tablets with release profiles as sustained as the ones described here should be retained in patient’s body to allow the drug to be absorbed. Accordingly, these systems can be ideal to develop floatable or sinkable tablets that can be retained in the stomach for longer periods of time [56]. 

**Figure 5:**
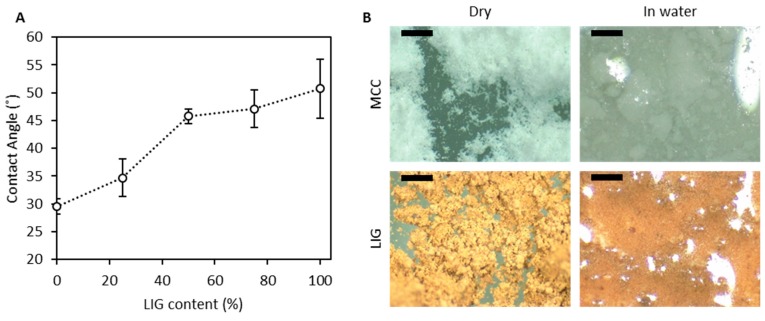
Static contact angle obtained for tablets using different concentrations of LIG and MCC (**A**). These tablets contain no drug. Pictures showing MCC and LIG powder dry and in water (**B**). The scale bar shows a distance of 0.5 mm.

The chemical nature of the excipients has a strong influence in the release profile of the tablets as can be seen in Figure 4C,D. Tablets with lower crushing strength are expected to show a quicker release profile [57]. However, in this case, tablets with higher LIG content showed the lower tensile strenght and the lower TC release. This can be explained by having a look at the nature of LIG. LIG is a hydrophobic molecule. Accordingly, tablets with higher LIG content will display certain degree of hydrophobicity. In order to evaluate this parameter, tablets were prepared using only MCC and LIG, and the static contact angle of water with their surfaces was evaluated (Figure 5A). It can be seen that as expected formulations with higher LIG content showed higher contact angle values. There was no significant difference between the obtained contact angle for tablets containing 0% and 25% of LIG (*p* = 0.371). On the other hand, tablets were more hydrophobic when the content of LIG was higher than 50% the (*p* < 0.05). Interestingly, there were no significant differences in the contact angles obtained for tablets containing 50%, 75% and 100% of LIG (*p* > 0.05).

This shows that the resulting tablets are not as wettable. Consequently, the access of water to the tablet will be more restricted in formulations containing higher LIG contents. Similar results were reported by Notley et al. for LIG and cellulose thin films [58]. Moreover, MCC shows a tendency to swell when immersed in water (Figure 5B) while LIG due to its hydrophobic nature did not show any swelling capabilities in water (Figure 5B). These factors can explain why formulations containing higher amounts of LIG presented lower TC releases. LIG did not swell when immersed in water. Due to its hydrophobicity, this biopolymer precludes tablet disintegration. Accordingly, this prevents TC release from the inner layers of the tablet. 

As mentioned before these results can be correlated with tablet disintegration. Figure S1 (Supporting information) shows images of the tablets at different times after placing them in water. It is obvious that tablets prepared using 5 tonnes of compression force are not disintegrating as easily as tablets prepared with lower compression forces. The “jumps” observed in the release profiles can be correlated with the fracture of the tablets when placed in water (Figure S1). L25 tablets fractured after 2 h while L55 tablets fractured at 3 h. On the other hand, it can be seen that L02 and L102 tablets did not disintegrate during the first 3 h while tablets containing LIG/MCC were totally disintegrated after 2 h. This is consistent with the release data that shows LIG/MCC tablets prepared using 2 tonnes showed higher TC release. Moreover, when LIG/PBS blends were incorporated into the formulation, the disintegration of the tablets is faster. Tablets containing only MCC or LIG as excipient take longer to start disintegrating. This is consistent with the dissolution profiles (Figure 4). 

As shown in Figure 5, MCC showed water uptake capacity while LIG did not. This gelation of MCC would explain the quick disintegration of MCC/LIG tablets. When MCC started to swell, this led to the disintegration of the tablet containing LIG as this molecule is hydrophobic (Figure 5). These findings are consistent with the ones reported by Pishnamazi et al. [43]. In this work, the use of lignin as excipient to prepare tablets containing acetylsalicylic acid is described. LIG containing tablets disintegrated faster than the ones that did not contain it. Moreover, this work showed faster dissolution profiles than the ones described in the present work. However, in this case the amount of drug was only a 5% (*w*/*w*) of the tablet and tablets contained disintegrants to make the release quicker. Finally, the tablets were more brittle than the ones described in the present work. This work study complements the one published by Pishnamazi et al. as it shows that LIG can be used as an excipient to prepare tablets with a wide variety of characteristics tailored to specific applications [43].

The overall results suggest that LIG is not an ideal excipient for tablet manufacturing when used in high concentrations and/or prepared using high compaction pressures as it leads to incomplete drug releases. However, when combined with MCC in combinations up to 1:1, it can be used to change the release properties of the tablets. It improves the disintegration of the tablets at shorter times and improves the amount of drug released.

### 3.4. Antioxidant Capabilities of LIG and MCC Blends

LIG is a well-known antioxidant compound [59,60,61]. This property could provide added value to the final product. It can be included as a protective agent that will prevent the degradation of drugs in the formulation.

**Figure 6:**
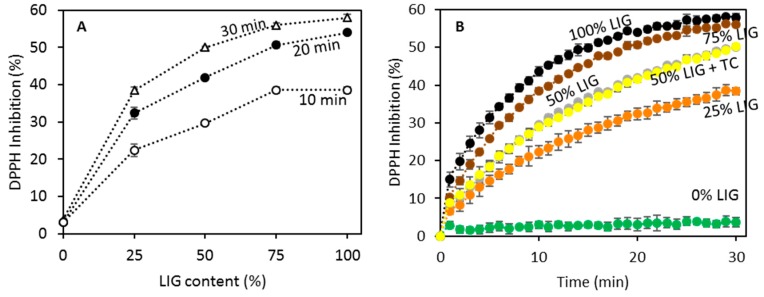
DPPH inhibition for LIG/MCC blends as a function of the LIG content for different incubation times (**A**). DPPH inhibition as a function of the incubation time for LIG/MCC dissolutions containing different amounts of LIG (**B**).

Figure 6 shows the results of the DPPH assay developed to ascertain the antioxidant capabilities of LIG/MCC blends. Figure 6A shows the antioxidant capabilities of the blends as a function of the incubation time in combination with DPPH. As expected, blends containing higher amounts of LIG showed higher antioxidant capacity. Moreover, formulations containing only MCC showed very low antioxidant capacity in comparison wo the other formulation. The antioxidant activity is affected by the incubation time (Figure 6A,B). Blends containing 75% of LIG showed similar antioxidant activity to pure LIG. The antioxidant capacity of LIG/MCC (50/50) and TC was measured to evaluate if there are synergistic effects as TC has been reported to have antioxidant activity in the past. However, in this case, we can see that the antioxidant activity of the formulation containing TC is equivalent to the equivalent formulation without TC (50% LIG). 

The antioxidant properties of different types of LIG have been proven in the past [26,49]. On the other hand, the antioxidant properties of MCC/LIG blends are lower than those of pure LIG. This is obvious, as MCC did not show any noticeable antioxidant activity (Figure 6). The incorporation of antioxidant excipients can be extremely beneficial for pharmaceutical formulations as they can provide protection to the active pharmaceutical ingredient [62]. The use of LIG as excipient can be extremely beneficial to be incorporated in dietary supplement tablets due to its antioxidant properties or to add value as a pharmaceutical excipient. Finally, it has been reported that LIG could provide benefits for human health, such as obesity control, antiviral activity, immunomodulation or anticoagulant activity [63]. 

## 4. Conclusions

In the present work, the use of LIG as excipient for tablet manufacturing via-direct compression has been studied. Studies describing pharmaceutical applications of LIG are scarce. In the present work, we demonstrated that LIG can be used to modify the dissolution profile of tablets when combined with MCC. By modifying different factors such as compaction pressure and LIG content, the release profile can be modified. Tablets made using LIG as the only excipient were fragile and were not able to release as much TC as the formulations prepared using LIG/MCC blends as excipient. However, the addition of smaller LIG quantities to the excipients can be used as a potential alternative to modify the drug release profile. Moreover, it was demonstrated that LIG/MCC had antioxidant activity that could play a crucial role to prevent the oxidation of the active ingredients in the formulation.

There is an urgent need to find renewable biopolymers that can substitute synthetic ones. The pharmaceutical industry extensively uses cellulose and cellulose derivatives that are natural biopolymers. However, there are a wide variety of synthetic pharmaceutical excipients used nowadays. Replacing all of them with natural ones is not possible due to certain limitations of natural biopolymers, such as their variability. However, LIG can be used as a pharmaceutical excipient for tablets not only for pharmaceutical applications. The scientific community is making extensive efforts to try to bring natural alternatives to cover this gap. Moreover, dietary supplements or even fertilizing containing tablets can be prepared using LIG as excipient. Consequently, LIG has proven to show interesting properties and, accordingly, we believe that its uses as pharmaceutical excipient should be exploited for different applications.

## Figures and Tables

**Table 1:** LIG and MCC densities, angle of repose, Hauser ratio and Carr index.

Material	Bulk Density (g/mL)	Tapped Density (g/mL)	Hausner Ratio	Carr Index	BET Specific Surface Area (m^2^/g)	Pore Size (Å)	Porosity
MCC	0.306 ± 0.002	0.348 ± 0.008	1.13 ± 0.02	12 ± 2	1.53	294	0.80 ± 0.02
LIG	0.354 ± 0.005	0.405 ± 0.005	1.14 ± 0.03	12 ± 2	5.45	238	0.74 ± 0.01

**Table 2:** Kawakita and Heckel Parameters calculated for MCC and LIG powders.

	Kawakita		Heckel	
	a	1/b (MPa)	P_γ_ (MPa)	D_a_
MCC	0.80 ± 0.01	2.3 ± 0.4	242 ± 32	0.88 ± 0.01
LIG	0.73 ± 0.01	7.9 ± 1.0	361 ± 77	0.78 ± 0.03

**Table 3:** Characteristics of prepared tablet formulations. The prepared tablets showed a diameter of 13.10 ± 0.01 mm.

Formulation	Compression (Tonnes/MPa)	Excipient Composition (%)		Thickness (mm)	Mass Uniformity (%)
		LIG	MCC		
L02	2.0/147.8	0.0	100.0	2.70 ± 0.02	0.5 ± 0.7
L22		25.0	75.0	2.74 ± 0.01	0.7 ± 0.4
L52		50.0	50.0	2.76 ± 0.01	1.1 ± 0.4
L72		75.0	25.0	2.82 ± 0.01	1.2 ± 0.3
L102		100.0	0.0	2.85 ± 0.02	6.5 ± 1.3
L05	5.0/369.4	0.0	100.0	2.56 ± 0.01	0.8 ± 0.3
L25		25.0	75.0	2.58 ± 0.02	1.1 ± 0.3
L55		50.0	50.0	2.61 ± 0.01	1.3 ± 0.3
L75		75.0	25.0	2.69 ± 0.01	1.9 ± 0.6
L105		100.0	0.0	2.72 ± 0.01	5.6 ± 2.7

## Supplementary Materials

The following are available online at https://www.mdpi.com/2218-273X/9/9/423/s1, Figure S1: Images of TC containing tablets at different times after placing them in water.
