# Supplementary material for: Lignin and Cellulose Blends as Pharmaceutical Excipient for Tablet Manufacturing via Direct Compression
Source: Biomolecules. 2019 Aug 28;9(9):423. doi: 10.3390/biom9090423 (PMC6770814; doi:10.3390/biom9090423)
Supplement: Supplementary file 1 [file biomolecules-09-00423-s001.pdf]

The influence of LIG on MCC gelling properties was evaluated. For this purpose, loaded tablets (containing tetracycline) applying compression forces of 2 and 5 tonnes were manufactured. Then, the tablets were placed in small containers containing 35 mL of deionized water and at defined points pictures were taken. The resulting images are shown in Figure S1.

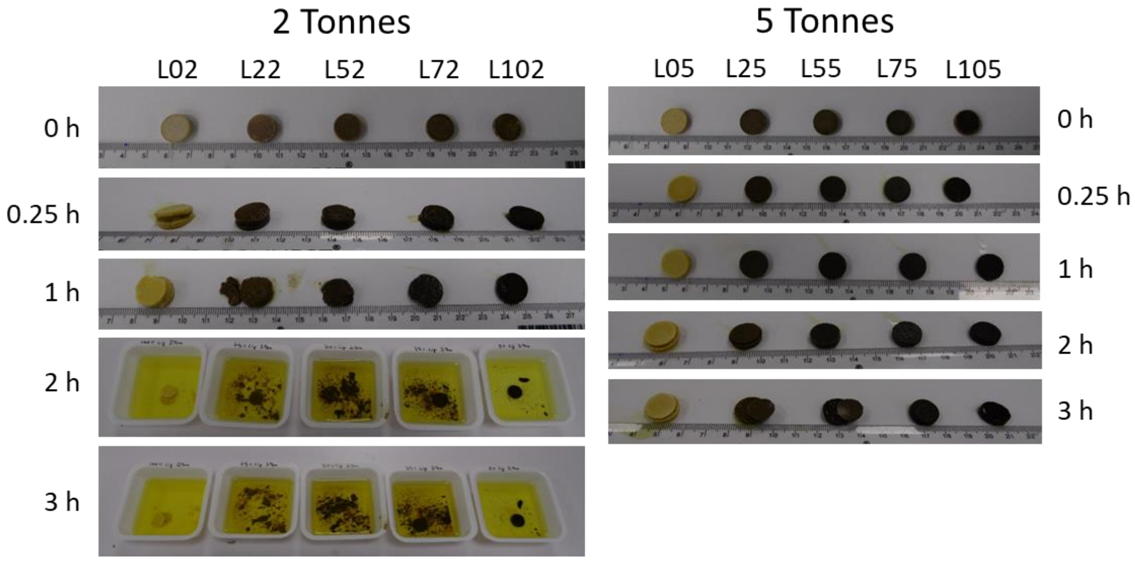

Figure S1. Images of TC containing tablets at different times after placing them in water.
